# Maternal Melatonin Programs the Daily Pattern of Energy Metabolism in Adult Offspring

**DOI:** 10.1371/journal.pone.0038795

**Published:** 2012-06-12

**Authors:** Danilo S. Ferreira, Fernanda G. Amaral, Caroline C. Mesquita, Ana Paula L. Barbosa, Camilo Lellis-Santos, Ariane O. Turati, Laila R. Santos, Carolina S. Sollon, Patricia R. Gomes, Juliana A. Faria, José Cipolla-Neto, Silvana Bordin, Gabriel F. Anhê

**Affiliations:** 1 Department of Pharmacology, Faculty of Medical Sciences, State University of Campinas, Campinas, Brazil; 2 Department of Physiology and Biophysics, Institute of Biomedical Sciences, University of Sao Paulo, Sao Paulo, Brazil; Vanderbilt University, United States of America

## Abstract

**Background:**

Shift work was recently described as a factor that increases the risk of Type 2 diabetes mellitus. In addition, rats born to mothers subjected to a phase shift throughout pregnancy are glucose intolerant. However, the mechanism by which a phase shift transmits metabolic information to the offspring has not been determined. Among several endocrine secretions, phase shifts in the light/dark cycle were described as altering the circadian profile of melatonin production by the pineal gland. The present study addresses the importance of maternal melatonin for the metabolic programming of the offspring.

**Methodology/Principal Findings:**

Female Wistar rats were submitted to SHAM surgery or pinealectomy (PINX). The PINX rats were divided into two groups and received either melatonin (PM) or vehicle. The SHAM, the PINX vehicle and the PM females were housed with male Wistar rats. Rats were allowed to mate and after weaning, the male and female offspring were subjected to a glucose tolerance test (GTT), a pyruvate tolerance test (PTT) and an insulin tolerance test (ITT). Pancreatic islets were isolated for insulin secretion, and insulin signaling was assessed in the liver and in the skeletal muscle by western blots. We found that male and female rats born to PINX mothers display glucose intolerance at the end of the light phase of the light/dark cycle, but not at the beginning. We further demonstrate that impaired glucose-stimulated insulin secretion and hepatic insulin resistance are mechanisms that may contribute to glucose intolerance in the offspring of PINX mothers. The metabolic programming described here occurs due to an absence of maternal melatonin because the offspring born to PINX mothers treated with melatonin were not glucose intolerant.

**Conclusions/Significance:**

The present results support the novel concept that maternal melatonin is responsible for the programming of the daily pattern of energy metabolism in their offspring.

## Introduction

Shift work, or unusual working schedules, is a working condition for approximately 20% of the working population in Europe and the U.S. [1]. A large body of epidemiological data clearly demonstrates that shift workers are predisposed to cardiovascular alterations, increased body mass index and obesity [2], [3], [4].

Recently, a large-scale study by Pan et al. revealed an increased risk for Type 2 diabetes mellitus (T2DM) in women exposed to an extended period of rotating night shift work [5].

In addition to the metabolic hazard to which shift workers are exposed, a recent investigation has revealed that the detrimental effects of this type of working schedule might influence the metabolism of their offspring. Varcoe and colleagues have demonstrated that rats born to mothers exposed to chronic phase shifts during pregnancy become glucose intolerant due to whole-body insulin resistance [6].

Apart from these convincing experimental and clinical reports relating phase shifts and shift work to an increased risk for T2DM, the mechanism by which circadian disruptions transmits information to the offspring remains unknown. Among several endocrine secretions, phase shifts or acute changes in the light/dark cycle were described as altering the circadian profile of melatonin in humans, mice and rats [7], [8], [9].

Melatonin, the main endocrine product of the pineal gland, is produced and secreted in a circadian fashion, peaking during the night [10]. It plays an important role as an interface for the cyclic environment, physiological rhythms, and neuroendocrine processes [11]. With regard to its metabolic activity, in vitro melatonin was reported to stimulate glucose uptake by adipocytes and muscle cells and to stimulate leptin expression [12], [13], [14]. Moreover, rats submitted to surgical ablation of the pineal gland become glucose intolerant and exhibit increased hepatic glucose production due to the absence of melatonin [15], [16].

The present study reveals that the removal of maternal melatonin during pregnancy and lactation by means of pinealectomy predisposes male and female offspring to glucose intolerance arising from hepatic insulin resistance and decreased insulin secretion.

## Materials and Methods

### Ethics Statement

All experiments were performed in accordance with the guidelines of the Brazilian College for Animal Experimentation (COBEA) and approved by the State University of Campinas Committee for Ethics in Animal Experimentation (Campinas, SP, Brazil).

### Animals, surgical procedures and treatments

Wistar rats (obtained from the Animal Breeding Center at the University of Campinas – Campinas, SP, Brazil) were used in all experiments and kept at 22°C±2°C. The light/dark cycle timepoints (ZT) were defined set to ZT24 (lights on) and ZT12 (lights off). Standard chow and water were provided ad libitum. Two-month old female rats were subjected to pinealectomy (PINX) or SHAM surgery as previously described [17]. Briefly, the rats were anesthetized with an i.p. injection of sodium thiopental (50 mg/kg body weight) and placed in a stereotaxic apparatus for small animals. A sagittal opening was made on the scalp, and the skin and muscles were pushed aside to expose the λ suture. By means of a circular drill, a disc-shaped perforation was made around the λ suture, and the disc-shaped piece of bone was delicately removed. Thereafter, the pineal gland (which is located just below the posterior venous sinus confluence) was removed with a fine forceps. The skull was closed by returning the disc-shaped bone, and the scalp was sutured with cotton thread. After all experiments, the effectiveness of the PINX was verified by a macroscopic *post-mortem* examination of the central nervous system to check for pineal absence.

One day after the surgical procedures, the PINX rats were further divided into two groups, receiving either vehicle or Melatonin (PM) diluted in the drinking water. Melatonin (Mel) supplementation was based on previous studies [18], [16]. Details for Mel supplementation are as follows: Melatonin (Sigma Chemical Company, St. Louis, MO, USA) was prepared twice a week by dissolving the Mel (50 mg) in ethanol (1 mL, 100%, v/v) and further dilution of this entire volume in 50 mL of distilled water. Based on the daily water intake and the body weight of each rat, the above mentioned solution of Mel (1 mg/mL) was further diluted into 200 mL of regular water so that night-time intake of Mel would be approximately 0.5 mg/kg. Body weight was acquired twice a week to adjust the dosage. Mean of the final concentration of Mel in the bottles was 0.005 mg/mL. Water bottles were covered with aluminium foil and made available to the rats at the moment of lights off (ZT12) and removed at the moment of lights on (ZT24). During the light phase of the light/dark cycle, bottles containing melatonin were replaced with bottles with regular water. The SHAM and PINX rats that did not receive Mel were supplemented with water containing ethanol (1×10^−8^% ethanol) exclusively between ZT12 and ZT24.

Thirty days after the surgery, the female rats were housed with one male rat for 5 days. The presence of spermatozoa in the vaginal lavage was checked daily to determine day 0 of gestation. The pregnant rats were immediately isolated in separate cages. On the day of the delivery, the number of pups was adjusted to 8 per lactating mother and the offspring were allowed to breast-feed until the 21^st^ day of life. Melatonin or vehicle supplementation was maintained throughout gestation and lactation.

The offspring born to the SHAM, PINX and PM mothers (thereafter designed as SHAM-P1, PINX-P1 and PM-P1, respectively) were separated from their mothers at the beginning of the 22^nd^ day after birth and adjusted to 4–5 animals per cage. At this time, the females were separated from the males. Thereafter, the offspring were kept at 22°C±2°C with lights on from 7:00 AM to 7:00 PM; standard chow and water were provided *ad libitum*. Except for fasting glucose levels and body weight registrations, siblings were not grouped together in any experimental protocol.

### Intraperitoneal pyruvate tolerance test (PTT)

The rats were fasted for 12 h, and a sodium pyruvate solution (250 mg/ml) was injected i.p. either at ZT3 (three hours of lights on) or at ZT10 (ten hours of lights on). The final dosage of pyruvate was 2 g/kg. Glucose was determined in the blood collected from the tail before (0 min) and 15, 30, 60, 90, 120 and 150 min after pyruvate injection. The area under the curve (AUC) of glycemia *vs.* time was calculated above each individual baseline (basal glycemia) to estimate the total glucose synthesized from pyruvate.

### Intraperitoneal glucose tolerance test (GTT)

The rats were fasted for 12 h prior to i.p. glucose injection (2 g/kg of a 20% solution of D-glucose) at ZT3 or ZT10. The blood samples were collected from the tail at 0, 10, 15, 30, 60 and 120 min for the measurement of blood glucose. The area under the curve (AUC) of glycemia *vs.* time was calculated above each individual baseline (basal glycemia) to estimate glucose tolerance.

### Intraperitoneal insulin tolerance test (ITT)

The rats were fasted for 12 h before the experiments prior to i.p. insulin injection (2 IU/kg), at ZT3 or ZT10. The blood samples were collected from the tail at 0, 5, 10, 15, 20, 25 and 30 min for the measurement of serum glucose. The constant rate for glucose disappearance (*K*_ITT_) was calculated using the formula 0.693/half-life. The glucose half-life was calculated from the slope of the least-squares analysis of the blood glucose concentrations during the linear phase of decay [19].

### Protein extraction and immunoblotting

The rats were anesthetized with sodium thiopental (5 mg/100 g body weight, i.p.) after a 12h-fatsing. The soleus muscle from the left paw was excised, the abdominal cavity was opened and a fragment of the liver was removed. These samples were used to assess basal tyrosine and AKT phosphorylation as well as the expression of PEPCK. Next, the *vena cava* was exposed and injected with 0.1 ml of a 10^−4^ M insulin solution. After 30 and 90 sec, an additional fragment of the liver and the remaining soleus were removed to assess insulin-stimulated tyrosine and AKT phosphorylation, respectively. The samples were processed for Western blotting as already reported [20]. In details, fragments of liver containing approximately 100 mg and the soleus muscles were homogenized in a boiling extraction buffer [10% sodium dodecyl sulfate, 100 mm Tris (pH 7.4), 10 mm EDTA, 10 mm sodium pyrophosphate, 100 mm sodium fluoride, 10 mm sodium vanadate] with a Polytron PTA 20S generator (model PT 10/35; Brinkmann Instruments, Inc., Westbury, NY) operated at maximum speed for 30 sec. The extracts were centrifuged at 15,000×g, 4 C, for 40 min to remove insoluble material. Protein concentrations of the supernatants were determined by the Bradford assay, and an equal amount of total protein from each sample (75 μg) was treated with Laemmli buffer containing dithiothreitol 100 mm. Samples were heated in a boiling water bath for 5 min, after which they were subjected to SDS-PAGE (10% bis-acrylamide). Electrotransfer of proteins from gel to nitrocellulose membrane was performed for 90 min at 120 V (constant) as described elsewhere. Nonspecific protein binding to nitrocellulose was reduced by preincubating the membrane overnight at 4 C in blocking buffer (5% non-fat dried milk, 10 mm Tris, 150 mm NaCl, and 0.02% Tween 20). The nitrocellulose membranes were incubated with the primary antibodies diluted in blocking buffer. The primary antibodies used were diluted as follows: anti-phospho-Tyr, anti-AKT1/2/3, anti-phospho-AKT1/2/3 (Ser473), anti-PEPCK, anti-IRS1, anti-IRS2 and anti-IR-β (Santa Cruz Biotechnology, Inc., Santa Cruz, CA). A secondary antibody conjugated with horseradish peroxidase (Bio-Rad, Hercules, CA) was used, followed by incubation with a solution containing Luminol, p-Coumaric Acid and H_2_O_2_. One minute after incubation, x-ray sensitive films were exposed to the membranes and marked by a chemiluminescent signal. Afterwards, membranes were reprobed with anti-GAPDH under the same conditions described above. Films were scanned and digital images were subjected to optical densitometry analysis using the software (Scion Corporation, Frederick, MD, USA). The signal obtained membrane incubated with one of the primary antibodies (Arbitrary Units) was normalized to the signal originated by the same membrane probed with anti-GAPDH antibody.

### Hormone measurements

Male *ad libitum*-fed rats were deeply anesthetized with sodium thiopental and decapitated for removal of trunk blood at ZT10. Serum was extracted and processed for leptin and insulin determination by ELISA according to the manufacturer's instructions (Cat No. EZRMI-13K and Cat No. EZRL-83K for insulin and leptin Kits, respectively; Merck Millipore, Billerica, MA, USA).

### Pancreatic islet isolation and insulin secretion

Five *ad libitum*-fed rats from each experimental group were anesthetized with sodium thiopental (5 mg/100 g body weight, i.p.) and decapitated at ZT10. Islets were isolated after perfusion and digestion of the pancreas with collagenase digestion solution [21]. After isolation, 12 groups of islets (five islets each) from each animal were incubated for 45 min at 37°C in Krebs–bicarbonate buffer containing 5.6 mM glucose and equilibrated with 95% O_2_/5% CO_2_, pH 7.4. The solution was then replaced with fresh Krebs–bicarbonate buffer, and the islets were incubated for 1 h with medium containing 5.6, 8.3, 11.1 or 16.7 mM glucose (Three groups per each glucose concentration). The incubation medium contained 115 mM NaCl, 5 mM KCl, 24 mM NaHCO_3_, 2.56 mM CaCl_2_, 1 mM MgCl_2_ and 0.3% BSA (w/v). The cumulative insulin release over 1 h was quantified in duplicate by radioimmunoassay using rat insulin as the standard [21].

### Statistical Analysis

The results are presented as the means ± SE. Comparisons were performed by two-way ANOVA, followed by Bonferroni post hoc testing (INStat; GraphPad Software, San Diego, CA). *P* values <0.05 indicate a significant difference.

## Results

### Pregnancy outcomes

We have found no difference in gestation length (21.5±0.8, 21.7±0.4 and 21.7±0.2 for SHAM, PINX and PM, respectively; P>0.05) and litter size (10.4±0.9, 10.0±0.6 and 10.5±0.7 for SHAM, PINX and PM, respectively; P>0.05) among the three experimental conditions.

### Absence of maternal melatonin during pregnancy and lactation does not change body weight and fasting glycemia in the offspring

**Figure 1:**
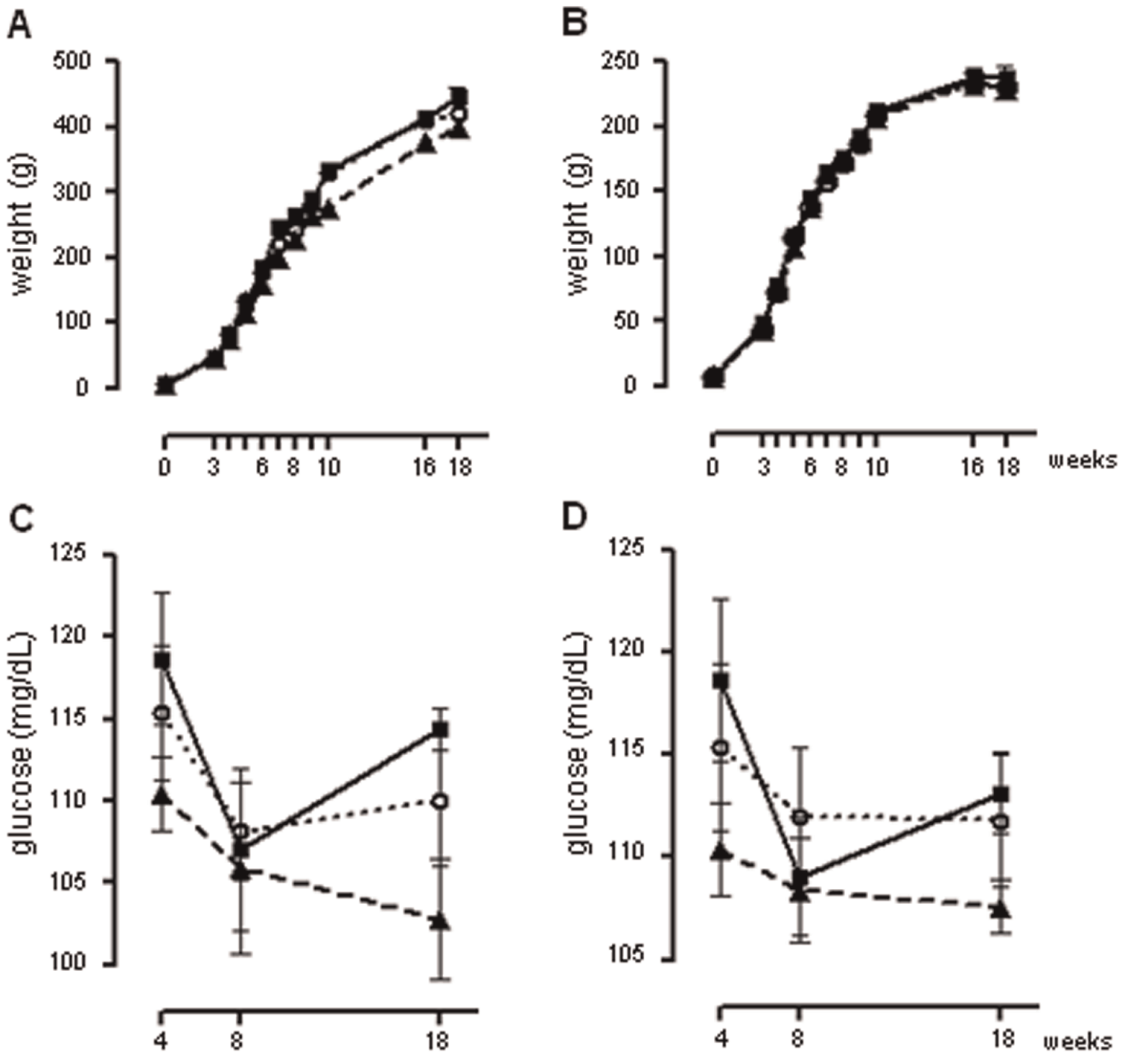
Body weight and basal glycemia in male and female SHAM-P1, PINX-P1 and PM-P1 rats. Male (**A**) and female (**B**) SHAM-P1, PINX-P1 and PM-P1 rats were weighed at the second day of life and at the end of the 3^rd^, 4^th^, 5^th^, 6^th^, 7^th^, 8^th^, 9^th^, 10^th^, 16^th^ and 18^th^ weeks of life at ZT10 (n=38 to 49 for males and n=35 to 41 for females). Glycemia of the male (**C**) and female (**D**) SHAM-P1, PINX-P1 and PM-P1 rats was assessed at the end of the 4^th^, 8^th^ and 18^th^ weeks of life at ZT10 after a 12h fasting (n=11 to 16 for males and n=9 to 12 for females). SHAM-P1 are dotted lines with open circles, PINX-P1 are continuous lines with closed squares and PM-P1 are dashed lines with closed triangles. The data are presented as the mean ± SE. *P<0.05 vs. week 0 within the same group.

Body weights from SHAM-P1, PINX-P1 and PM-P1were always assessed at ZT10. Changes in body weight from the first day of life to the 18^th^ week were similar among male and female SHAM-P1, PINX-P1 and PM-P1 rats (Fig. 1A and 1B, respectively). Glycemia measured at ZT10 after a 12h-fasting (two hours before lights off) were also similar among male and female SHAM-P1, PINX-P1 and PM-P1 rats at the 4^th^, 8^th^, 16^th^ and 18^th^ weeks of life (Fig. 1C and 1D). Adiposity of female SHAM-P1, PINX-P1 and PM-P1presented similar values at the 16^th^ week of life at ZT10. In addition, circulating leptin levels of male SHAM-P1, PINX-P1 and PM-P1were also similar at the 16^th^ week of life at ZT10 (Figure S1).

### Absence of maternal melatonin during pregnancy and lactation causes glucose intolerance in the offspring

**Figure 2:**
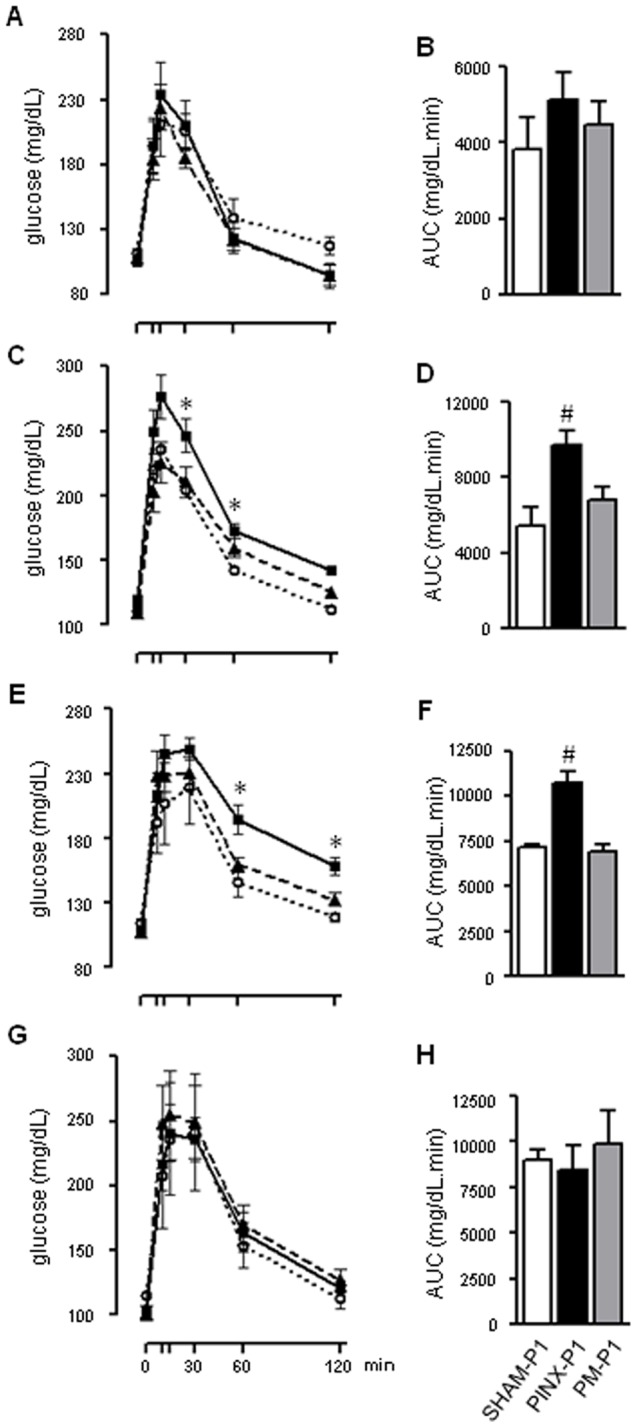
Glucose tolerance in male and female SHAM-P1, PINX-P1 and PM-P1 rats at different moments of the light/dark cycle. Fasting glycemia was assessed in the male and female SHAM-P1, PINX-P1 and PM-P1 rats. Next, the animals received an i.p. injection of glucose (2 g/kg), and glycemia was assessed at 10, 15, 30, 60 and 120 min after injection. The glycemic values were plotted vs. time after the injection, and the AUC was calculated. Tests were performed in male offspring at the end of the 8^th^ (**A** and **B**) and 18^th^ (**C** and **D**) weeks of life at ZT10. Tests were also performed in female offspring at the end of the 18^th^ week of life at ZT10 (**E** and **F**) and in male offspring at the end of the 18^th^ week of life at ZT3 (**G** and **H**). SHAM-P1 are dotted lines with open circles, PINX-P1 are continuous lines with closed squares and PM-P1 are dashed lines with closed triangles. The data are presented as the mean ± SE. *P<0.05 vs. SHAM-P1 at the same time after glucose injection; #P<0.05 vs. SHAM-P1 (n=6).

Our first approach to seek for metabolic changes in the offspring consisted of GTT performed in the 8-week old male offspring. This test was performed at ZT10 because this is a key moment in the light/dark cycle at which time adult PINX rats display glucose intolerance [15]. In comparing the SHAM-P1, PINX-P1 and PM-P1 rats, we found no differences in glucose tolerance (Fig. 2A and 2B).

To verify if metabolic alterations would manifest later in the adult life of the PINX-P1 rats, we performed a GTT at the 18^th^ week of life. These tests were initially performed at ZT10 and revealed that the PINX-P1 rats were glucose intolerant. This result was more clearly observed at 30 and 60 min after the glucose injection, when blood glucose levels in the male PINX-P1 rats were 20% and 22% higher, respectively, than those in the male SHAM-P1 rats (P<0.05). The male PM-P1 rats had blood glucose levels similar to those of the male SHAM-P1 rats throughout the entire GTT (Fig. 2C). The AUC values obtained from the GTT results for the male PINX-P1 rats, but not those for the male PM-P1 rats, were 78% higher than the values for the SHAM-P1 males (P<0.05) (Fig. 2D).

Glucose intolerance was also observed in the female PINX-P1 rats, based on the GTT results at ZT10. This result was more clearly observed at 60 and 120 min after the glucose injection, when the blood glucose levels for the female PINX-P1 rats were 33% and 30% higher, respectively, than the SHAM-P1 females (P<0.05). The female PM-P1 rats also had blood glucose levels similar to those of the SHAM-P1females throughout the entire GTT (Fig. 2E). Concordantly, the AUC values obtained from the GTT results for the female PINX-P1 rats, but not those obtained for the female PM-P1 rats, were 77% higher than the values for the SHAM-P1 females (P<0.05) (Fig. 2F).

In contrast to the observations at ZT10 from both male and female offspring, a GTT performed at ZT3 (three hours after lights on) revealed that the male PINX-P1 rats were not glucose intolerant at this time-point of the light/dark cycle (Fig. 2G and 2H).

### Absence of maternal melatonin during pregnancy and lactation does not cause whole-body insulin resistance in the offspring

**Figure 3:**
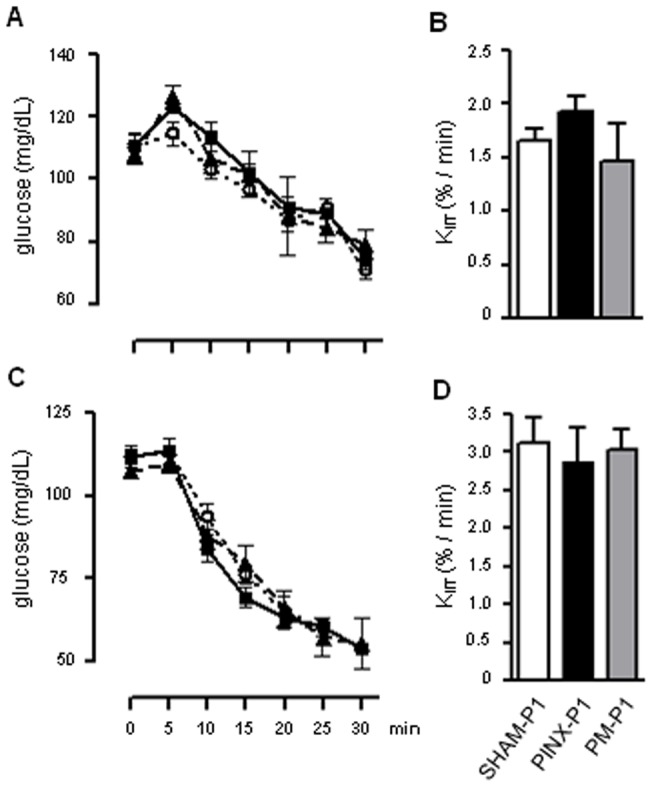
Whole body insulin sensitivity in male and female SHAM-P1, PINX-P1 and PM-P1 rats. Fasting glycemia was assessed in male and female SHAM-P1, PINX-P1 and PM-P1 rats. Next, rats received an i.p. injection of insulin (2 IU/kg), and glycemia was assessed at 5, 10, 15, 20, 25 and 30 min after injection. The glycemic values were plotted vs. time after the injection, and the K_ITT_ was calculated. Tests were performed in male (**A** and **B**) and female (**C** and **D**) offspring at the 18^th^ week of life at ZT10. SHAM-P1 are dotted lines with open circles, PINX-P1 are continuous lines with closed squares and PM-P1 are dashed lines with closed triangles. The data are presented as the mean ± SE (n=6).

To seek a mechanism by which glucose intolerance occurs in the PINX-P1 rats, we next performed ITT in both male and female SHAM-P1, PINX-P1 and PM-P1 rats. The ITT revealed that whole-body insulin resistance had not developed in the male PINX-P1 rats. This finding was evidenced by similar glucose levels after the insulin injection (Fig. 3A) and similar K_ITT_ values (Fig. 3B) compared to the SHAM-P1 rats. Similar results were found in the female offspring (Fig. 3C and 3D).

### Absence of maternal melatonin during pregnancy and lactation increases whole-body gluconeogenesis in the offspring

**Figure 4:**
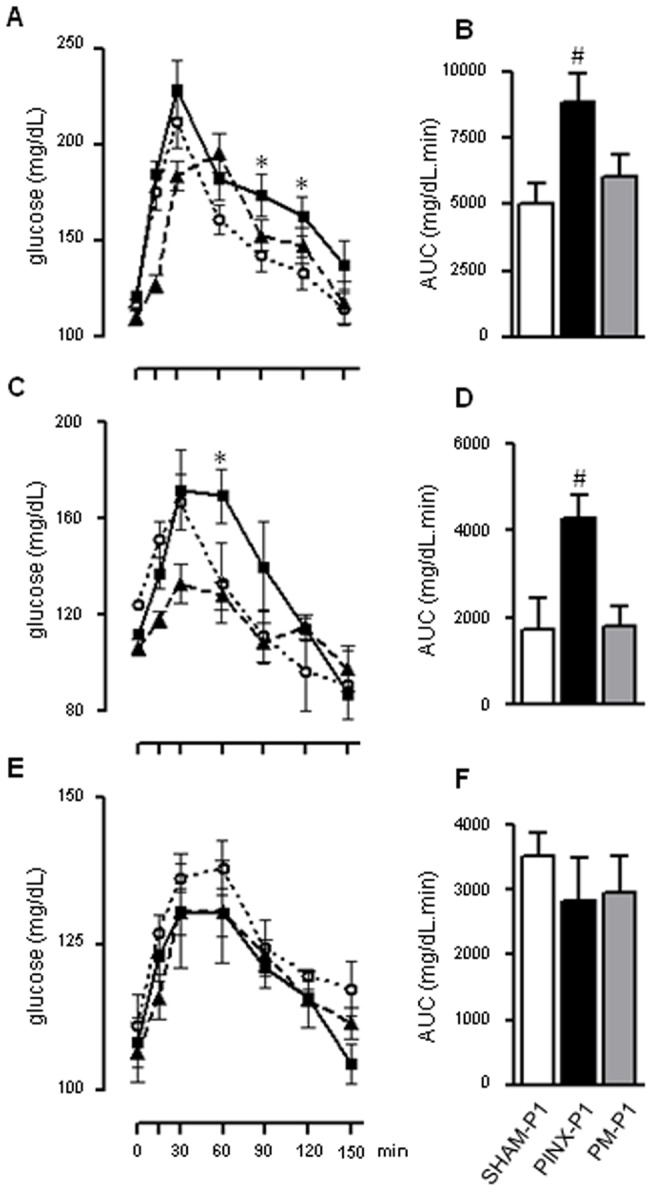
Glucose production from pyruvate in male and female SHAM-P1, PINX-P1 and PM-P1 at different moments of the light/dark cycle. Fasting glycemia was assessed in male and female SHAM-P1, PINX-P1 and PM-P1 rats. Next, the rats received an i.p. injection with sodium pyruvate (2 g/kg), and glycemia was assessed at 15, 30, 60, 90, 120 and 150 min after injection. The values of glycemia were plotted vs. time after injection, and the AUC was calculated. Tests were performed in male (A and B) and female (C and D) offspring at the 18^th^ week of life at ZT10. Tests were also performed in male offspring at 18 weeks of life at ZT3 (E and F). SHAM-P1 are dotted lines with open circles, PINX-P1 are continuous lines with closed squares and PM-P1 are dashed lines with closed triangles. The data are presented as the mean ± SE. *P<0.05 vs. SHAM-P1 at the same time after pyruvate injection; #P<0.05 vs. SHAM-P1 (n=6).

In addition to a reduction in whole-body glucose disposal (mainly accounted for by skeletal muscle glucose uptake), increased hepatic glucose production (HGP) has also been reported to contribute to glucose intolerance [22]. To assess whether increased HGP was involved in the glucose intolerance of the PINX-P1 rats, we performed a PTT in the SHAM-P1, PINX-P1 and PM-P1 rats at ZT10. The PTT measures the extension of the whole-body conversion of pyruvate into glucose and allows the assessment of the rate of gluconeogenesis, which is an important component of HGP. We found increased glucose levels after pyruvate injection in the male PINX-P1 rats at ZT10. This result was more clearly observed 90 and 120 min after the pyruvate load, when the blood glucose levels were 28% and 27% higher, respectively, than in the male SHAM-P1 rats (P<0.05). Similarly to the GTT results, the male PM-P1 rats had blood glucose levels similar to those of the male SHAM-P1 rats throughout the entire PTT (Fig. 4A). The AUC values obtained for the PTT of the male PINX-P1 rats, but not those for the male PM-P1 rats, were 75% higher than for the male SHAM-P1 rats (P<0.05) (Fig. 4B).

We also found increased gluconeogenesis in the female PINX-P1 rats based on the PTT results at ZT10. This finding was more clearly observed 60 min after the pyruvate injection, when the blood glucose levels of the female PINX-P1 rats were 27% higher than for the female SHAM-P1 rats (P<0.05). The female PM-P1 rats had blood glucose levels similar to those of the female SHAM-P1 rats throughout the entire PTT (Fig. 4C), and the AUC values obtained for the PTT of the female PINX-P1 rats, but not the female PM-P1 rats, were 144% higher than those for the female SHAM-P1 rats (P<0.05) (Fig. 4D).

In agreement with our GTT data, we found an unaltered response to the PTT performed at ZT3 in the male PINX-P1 rat compared to the male SHAM-P1 rats (Fig. 4E and 4F).

### Absence of maternal melatonin during pregnancy and lactation reduces glucose-stimulated insulin secretion in the offspring

**Figure 5:**
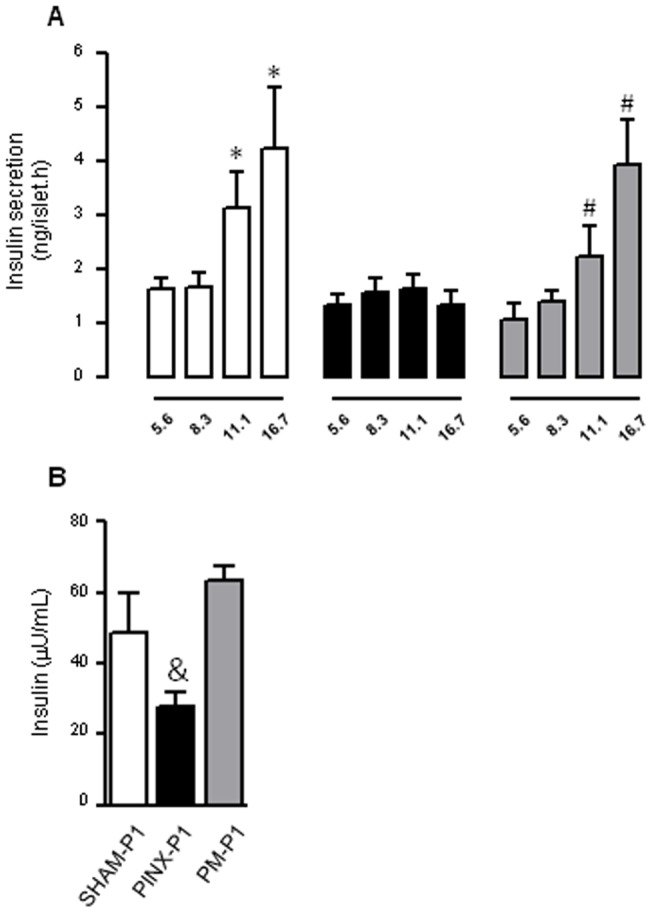
Insulin levels and insulin secretion by pancreatic islets of male SHAM-P1, PINX-P1 and PM-P1 rats. Male offspring were decapitated at ZT10, the pancreata were perfused with collagenase and the islets were isolated. After isolation, groups of five islets were initially incubated for 45 min at 37°C in Krebs–bicarbonate buffer containing 5.6 mM glucose and equilibrated with 95% O_2_–5% CO_2_, pH 7.4. The solution was then replaced with fresh Krebs–bicarbonate buffer, and the islets were incubated for an additional 1 h with medium containing 5.6, 8.3, 11.1 or 16.7 mM glucose. Insulin was quantified by RIA in the supernatant (**A**). Serum from the rats was used to determine circulating insulin levels (**B**). Open bars are SHAM-P1, black bars are PINX-P1 and grey bars are PM-P1. The data represent the cumulative insulin secretion over 1 h and are given as the mean ± SE. *P<0.05 vs. SHAM-P1 with 5.6 mM glucose; #P<0.05 vs. PM-P1 with 5.6 mM glucose; &P<0.05 vs. SHAM-P1 (n=5 for insulin secretion and insulin levels).

To extend our knowledge regarding glucose intolerance in the PINX-P1 rats, we next performed insulin secretion experiments using islets isolated from the male offspring at ZT10. We detected the expected increase in insulin secretion by the islets isolated from the SHAM-P1 rats incubated with stimulatory glucose concentrations. In this case, we found that 11.1 and 16.7 mM glucose stimulated insulin secretion to values that were 91% and 151% higher, respectively, than 5.6 mM glucose (P<0.05). Insulin secretion by islets isolated from the PINX-P1 rats was similar to that of the SHAM-P1 rats when incubated with 5.6 mM glucose. However, the islets from the PINX-P1 rats failed to increase insulin secretion when challenged with 11.1 and 16.7 mM glucose. The impaired response to glucose in the islets of the PINX-P1 rats was absent in the islets of the PM-P1 rats (Fig. 5A).

In accordance with the data from isolated islets, we have found that insulin levels of male PINX-P1 rats at ZT10 were 57% lower than those of SHAM-P1 (P<0.05). This adaptation was not found in PM-P1 rats (Fig. 5B).

### Absence of maternal melatonin during pregnancy and lactation impairs insulin signaling in the livers of the offspring

To evaluate the molecular events leading to hepatic insulin resistance in the PINX-P1 rats, we assessed the activity status of the insulin signaling steps that are pivotal to the control of hepatic gluconeogenesis.

The canonical early steps activated by insulin in peripheral tissues are tyrosine phosphorylation of pp95, mainly composed of the β subunit of the insulin receptor (IR-β), and pp185, mainly composed of the insulin receptor substrate 1 and 2 (IRS1 and 2) [23], [24]. Insulin-induced hepatic tyrosine phosphorylation of pp95 and pp185 at ZT10 was similar in the male SHAM-P1, PINX-P1 and PM-P1 rats. We also found no changes in IR-β, IRS1 and IRS2 expression in the livers of the male PINX-P1 and PM-P1 rats compared to the male SHAM-P1 rats (Figure S2).

**Figure 6:**
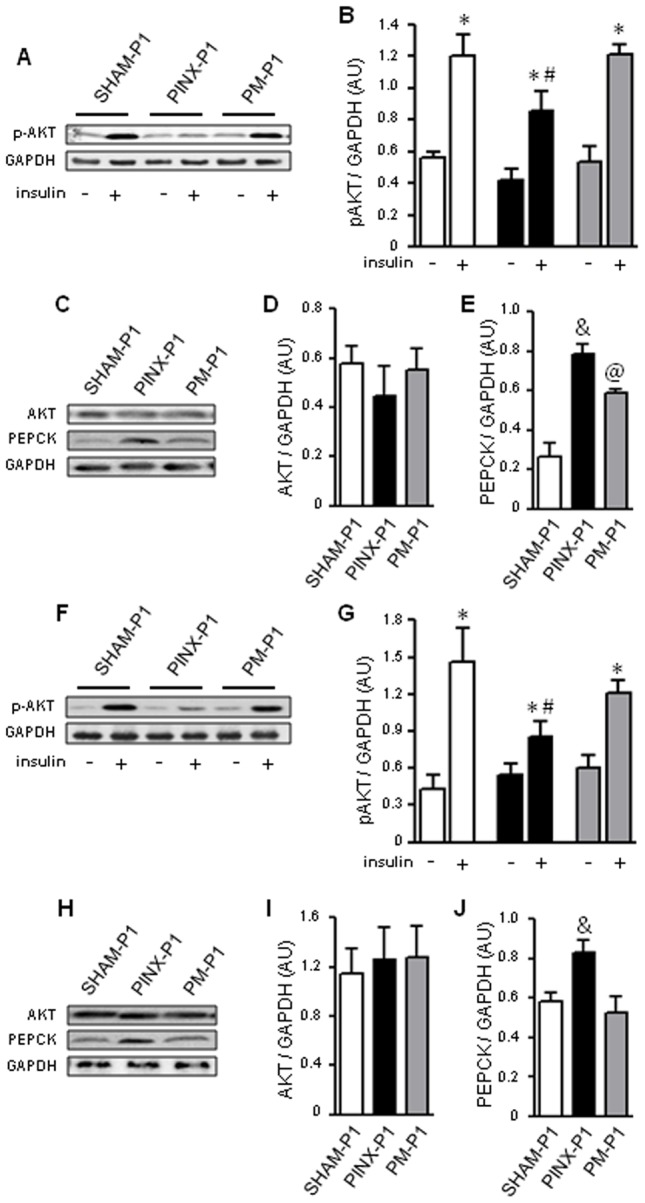
Insulin signaling in the livers of male and female SHAM-P1, PINX-P1 and PM-P1 rats. Male and female offspring at the 18^th^ week of life were anesthetized at ZT10, and a fragment of the liver was removed to detect basal phosphorylation. An additional fragment of the liver was removed 30 seconds after an intravenous insulin injection. The samples were processed by protein extraction and western blot detection of pAKT and GAPDH in male (**A**) and in female (**F**) offspring. The values obtained from male and female pAKT were normalized to GAPDH (**B** and **G**, respectively). The samples used for basal phosphorylation were run on a separate gel and transferred to membranes for the western blot detection of AKT, PEPCK and GAPDH in male (**C**) and in female (**H**) offspring. The values of AKT and PEPCK obtained from male (**D** and **E**, respectively) and from female offspring (**I** and **J**, respectively) were normalized to GAPDH. Open bars are SHAM-P1, black bars are PINX-P1 and grey bars are PM-P1. The data are presented as the mean ± SE. *P<0.05 vs. non-stimulated within the same group; #P<0.05 vs. insulin-stimulated SHAM-P1; &P<0.05 vs. non-stimulated SHAM-P1; @P<0.05 vs. non-stimulated PINX-P1 (n=6).

AKT serine phosphorylation is a critical node in insulin signaling downstream of IRS1/2 tyrosine phosphorylation and plays a key role in the suppression of gluconeogenesis [25]. Our experiments show that insulin efficiently increased hepatic AKT phosphorylation in the male SHAM-P1, PINX-P1 and PM-P1 rats. However, insulin-stimulated AKT phosphorylation in the livers of the PINX-P1 rats was 33% lower than in the livers of the SHAM-P1 rats. The impairment of insulin-induced AKT phosphorylation was not detected in the livers of the PM-P1 rats (Fig. 6B). No changes in AKT expression were detected among the groups (Fig. 6D).

Phosphoenol pyruvate carboxy kinase (PEPCK) is one limiting step for hepatic gluconeogenesis that are suppressed by insulin upon AKT activation [25]. We found that PEPCK expression was higher in the livers of the male PINX-P1 rats (199% higher than in the male SHAM-P1 rats; P<0.05). Hepatic PEPCK levels in the PM-P1 rats were higher than in the SHAM-P1 rats, but 25% lower than in the PINX-P1 rats (P<0.05) (Fig. 6E).

Insulin signaling in the livers of the female PINX-P1 rats was also modulated. Similar to the male offspring, the female PINX-P1 rats displayed unaltered insulin-induced tyrosine phosphorylation of pp185 and pp95 compared to the female SHAM-P1 and PM-P1 rats. IR-β, IRS1 and IRS2 expression in the livers of the female offspring were also not modulated by the absence of melatonin during gestation and lactation (Figure S3). Insulin-induced AKT phosphorylation was reduced in the livers from female PINX-P1 rats (42% lower than in the insulin-stimulated female SHAM-P1 rats, P<0.05). This reduction was not observed in the female PM-P1 rats (Fig. 6G). No changes in AKT expression were detected among the groups (Fig. 6I). PEPCK expression was increased in the livers from the female PINX-P1 rats (30% higher than female SHAM-P1, P<0.05) but not in the female PM-P1 rats (Fig. 6J).

### Absence of maternal melatonin during pregnancy and lactation does not alter insulin signaling in the skeletal muscle of the offspring

**Figure 7:**
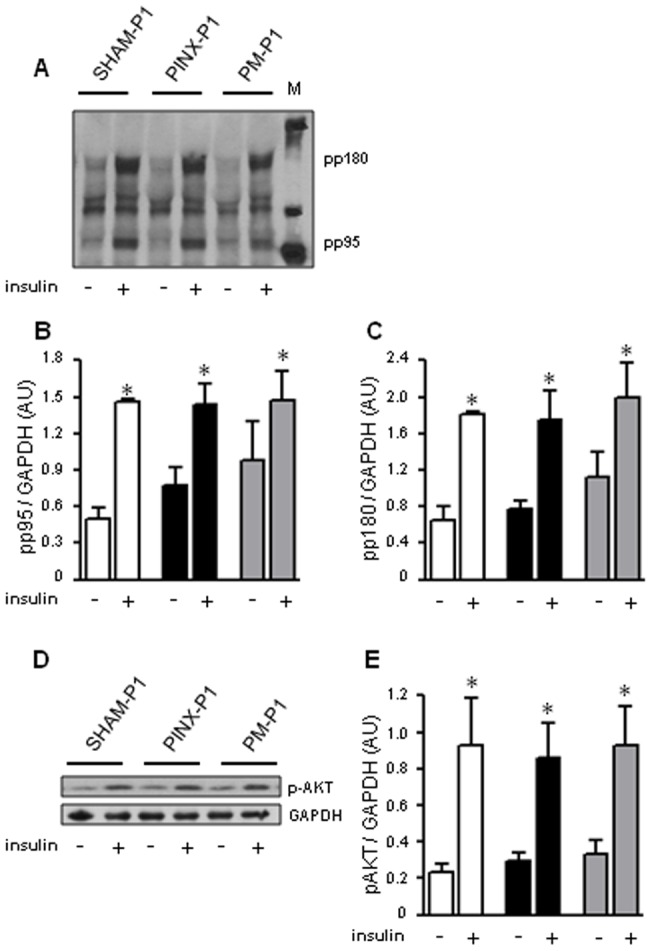
Insulin signaling in the skeletal muscle of male SHAM-P1, PINX-P1 and PM-P1 rats. Male offspring at the 18^th^ week of life were anesthetized at ZT10, and a soleus skeletal muscle was removed to detect basal phosphorylation. The remaining soleus muscle was removed 90 seconds after an intravenous insulin injection. The samples were processed by protein extraction and western blot detection of p-Tyr (**A**), pAKT and GAPDH (**D**). The values for tyrosine-phosphorylated pp95 (**B**) and pp185 (**C**) and pAKT (**E**) were normalized to GAPDH. Open bars are SHAM-P1, black bars are PINX-P1 and grey bars are PM-P1. The data are presented as the mean ± SE. *P<0.05 vs. non-stimulated within the same group (n=6).

We have also assessed insulin signaling in the skeletal muscle of the male PINX-P1 rats at ZT10. Insulin-stimulated tyrosine phosphorylation of pp95 and pp185 and serine phosphorylation of AKT were not altered in the skeletal muscle of the PINX-P1 rats compared to the SHAM-P1 rats (Fig. 7B, 7C and 7E, respectively). IR-β, IRS1, IRS2 and AKT expression in the skeletal muscle were similar among the male SHAM-P1, PINX-P1 and PM-P1 rats (Figure S4).

## Discussion

Increased pineal gland secretory activity during pregnancy is recognized as accounting for the high levels of nighttime circulating melatonin at the end of the gestational period in rats [26]. Maternal treatment with melatonin during pregnancy has been described to recover birth weight in rats born to undernourished mothers and to protect the placenta from ischemia/reperfusion-induced oxidative stress [27], [28]. Apart from this, the present study is the first to show that maternal melatonin has long-term effects in offspring by modulating glucose homeostasis. The present data are of particular importance because life-style and working schedules associated with disruptions in melatonin secretion are highly prevalent in developed countries [1], [29], [30].

Chronic shift work, in particular, was recently described to increase T2DM risk [5] and to display an inverse correlation with melatonin production due to light exposure at night [31]. In addition, Varcoe et al. recently demonstrated that a light phase shift during pregnancy is associated with glucose intolerance in offspring [6]. Thus, the present data favor the proposition that changes in photoperiod cycling might extend a detrimental effect on glucose homeostasis to the offspring, due to disruption of melatonin secretion during pregnancy. Glucose intolerance in the offspring of rats born to PINX mothers, however, was detected at the 18^th^ week of life. This is earlier than the glucose intolerance found by Varcoe et al. in rats born to mothers exposed to a phase shift (12 months). It is possible that this premature response occurs due to a significant absence of maternal melatonin due to pineal ablation. Phase shifts, in turns, may only impair, rather than suppress, melatonin secretion [7], [8], [9].

Several studies have demonstrated that the surgical removal of the pineal gland causes glucose intolerance due to the absence of melatonin [16], [32]. Our results show that rats born to PINX mothers become glucose intolerant due to the absence of maternal melatonin because its reposition throughout pregnancy and lactation renders the offspring from PINX mothers not glucose intolerant. Interestingly, adult PINX rats display glucose intolerance at the end of the light phase of the light/dark cycle [15]. We observed this same characteristic in rats born to PINX mothers. Thus, the absence of maternal melatonin imprints a metabolic dyssynchrony in the offspring that is similar to that found in adult PINX rats.

Our results also show that the offspring born to PINX mothers become glucose intolerant in part due to hepatic insulin resistance and increased gluconeogenesis. In addition, insulin-induced AKT phosphorylation, a crucial step for the repression of gluconeogenesis by insulin, is abrogated in the liver of offspring born to PINX mothers. The mechanism by which insulin-stimulated AKT phosphorylation suppresses hepatic gluconeogenesis relies on the inhibition of PEPCK expression. Accordingly, we have found increased PEPCK expression in the livers of rats born to PINX mothers.

The hepatic insulin resistance found in rats born to PINX mothers shares similarities with the previously described adult PINX rats. The adult PINX rats, as well as the offspring born to PINX mothers, display decreased insulin-induced AKT phosphorylation and increased PEPCK expression and gluconeogenesis. In the case of the adult rat without the pineal gland, hepatic insulin resistance occurs due to upregulation of TRB3, a pseudo-kinase inhibitor of AKT [16]. A mechanism that causes hepatic insulin resistance by the direct targeting of AKT may also occur in the offspring born to PINX mothers because no changes in the steps upstream of AKT, such as IR and IRS tyrosine phosphorylation, were detected.

Apart from the hepatic insulin resistance and increased gluconeogenesis, the mechanisms leading to glucose intolerance in offspring born to PINX mothers did not fully mirror those of PINX adult rats. PINX rats have a well-characterized whole-body insulin resistance, with reduced K_ITT_ values and decreased expression of the insulin-sensitive glucose transporter GLUT4 in the skeletal muscle [32]. Offspring born to PINX mothers have an unaltered glucose disappearance rate after insulin challenge. This finding suggest that rats born from mothers without melatonin during pregnancy and lactation do not develop insulin resistance in the skeletal muscle, given that this territory contributes up to 80% of the whole-body glucose disposal induced by insulin [33]. In support of this proposition, we have found no changes in insulin-induced phosphorylation of IRS1/2 and AKT in the skeletal muscle of offspring born to PINX mothers. Importantly, IRS and AKT phosphorylation are critical steps required for insulin-stimulated glucose uptake by muscle cells [34], [35].

Another endocrine feature that differs in the offspring of PINX mothers compared to the PINX rats is the pattern of insulin secretion by isolated pancreatic islets. Glucose-stimulated insulin secretion from PINX rats was upregulated at different moments of the light/dark cycle [36]. In fact, melatonin was described to act directly in the pancreatic β-cell to inhibit insulin secretion [37], [38]. In contrast, the present data demonstrate that glucose-stimulated insulin secretion from the offspring born to PINX mothers is actually lower than in offspring born to SHAM mothers at ZT10. Concordantly, insulin levels were reduced in PINX-P1, but not in PM-P1 rats at ZT10. The impaired secretory response of the pancreatic islets of the PINX-P1 animals is consistent with the information that melatonin activates signaling pathways, such as PI3K/AKT and MERK/ERK, that are implicated in the control of apoptosis and the proliferation of pancreatic β-cells [39]. It is possible that the premature absence of melatonin results in reduced activation of these pathways in the pancreatic islets of the fetal/newborn rat, thus impacting its adult phenotype.

Although the description of the mechanism by which the absence of gestational melatonin programs glucose intolerance in the offspring is not within the framework of the present investigation, increased corticosterone levels in early life of rats born to PINX mothers is a likely mechanism. It was previously demonstrated that primates born to pregnant mother exposed to continuous light exhibit increased cortisol levels at the 4^th^ day of life. This endocrine programming was not seen when mothers exposed to continuous light were supplemented with melatonin [40]. Additional studies have demonstrated that maternal melatonin actually suppresses ACTH-induced cortisol production through a mechanism dependent on the activation of MT1/2 receptors located in the adrenal gland [41]. In addition to these data based on primate adrenal gland, melatonin was demonstrated to inhibit the expression of steroidogenic acute regulatory protein (StAR), a limiting step for glucocorticoid synthesis, in cultured fetal adrenal glands isolated from rats [42]. Noteworthy, fetal exposure to glucocorticoid programs increased PEPCK expression and glucose intolerance in the adult offspring [43].

In conclusion, we have shown that the absence of maternal melatonin during pregnancy and lactation programs the offspring to glucose intolerance mainly due to hepatic insulin resistance and decreased insulin secretion. These data reveal a yet unknown role for melatonin in animal physiology that comprises a putative mechanism by which chronic phase shift during pregnancy disrupts energy homeostasis in the offspring. In addition, the present investigation provides basic information that supports epidemiological studies focusing on the offspring born to mothers subjected to light exposure during the night, such as night shift working schedules and the contemporary phenomenon of light pollution during the evening/night hours.

## Supporting Information

Figure S1 **Adiposity and serum leptin levels in SHAM-P1, PINX-P1 and PM-P1 rats.** Female offspring were anesthetized and weighted at ZT10. Periovarian fat pads were removed and weighted. Adiposity was expressed as the periovarian fat pad mass relative to the rat mass (**A**). Male rats were anesthetized and decapitated for removal of trunk blood at ZT10. Serum was extracted and processed for leptin determination by ELISA (**B**). Open bars are SHAM-P1, black bars are PINX-P1 and grey bars are PM-P1. Data are presented as mean ± SE (N=4 to 7 for adiposity; N=5 for leptin measurement).
(DOC)

Figure S2 **Insulin signaling in liver of male SHAM-P1, PINX-P1 and PM-P1.** Male offspring were anesthetized at ZT10 and a fragment of the liver was removed to detected basal phosphorylation. An additional fragment of the liver was removed 30 seconds after an intravenous insulin injection. Samples were processed protein extraction and western blot detection of pTyr (**A**), IR-β, IRS1, IRS2 and GAPDH (**D**). Values of tyrosine phosphorylated pp95 (**B**) and pp185 (**C**) and IR-β (**E**), IRS1 (**F**) and IRS2 (**G**) were normalized to GAPDH Open bars are SHAM-P1, black bars are PINX-P1 and grey bars are PM-P1. Data are presented as mean ± SE. *P<0.05 vs. non-stimulated within the same group (N=6).
(DOC)

Figure S3 **Insulin signaling in liver of female SHAM-P1, PINX-P1 and PM-P1.** Female offspring were anesthetized at ZT10 and a fragment of the liver was removed to detected basal phosphorylation. An additional fragment of the liver was removed 30 seconds after an intravenous insulin injection. Samples were processed protein extraction and western blot detection of pTyr (**A**), IR-β, IRS1, IRS2 and GAPDH (**D**). Values of tyrosine phosphorylated pp95 (**B**) and pp185 (**C**) and IR-β (**E**), IRS1 (**F**) and IRS2 (**G**) were normalized to GAPDH Open bars are SHAM-P1, black bars are PINX-P1 and grey bars are PM-P1. Data are presented as mean ± SE. *P<0.05 vs. non-stimulated within the same group (N=6).
(DOC)

Figure S4 **Insulin signaling in skeletal muscle of male SHAM-P1, PINX-P1 and PM-P1.** Male offspring were anesthetized at ZT10 and a soleus skeletal muscle was removed and processed protein extraction and western blot detection of IR-β, IRS1, IRS2, AKT and GAPDH (**A**). Values of IR-β (**B**), IRS1 (**C**), IRS2 (**D**) and AKT (**E**) were normalized to GAPDH. Open bars are SHAM-P1, black bars are PINX-P1 and grey bars are PM-P1. Data are presented as mean ± SE (N=6).
(DOC)
