# Supplementary figures and images for: Maternal Melatonin Programs the Daily Pattern of Energy Metabolism in Adult Offspring
Source: PLoS One. 2012 Jun 12;7(6):e38795. doi: 10.1371/journal.pone.0038795 (PMC3373595; doi:10.1371/journal.pone.0038795)

**Figure S1**

**
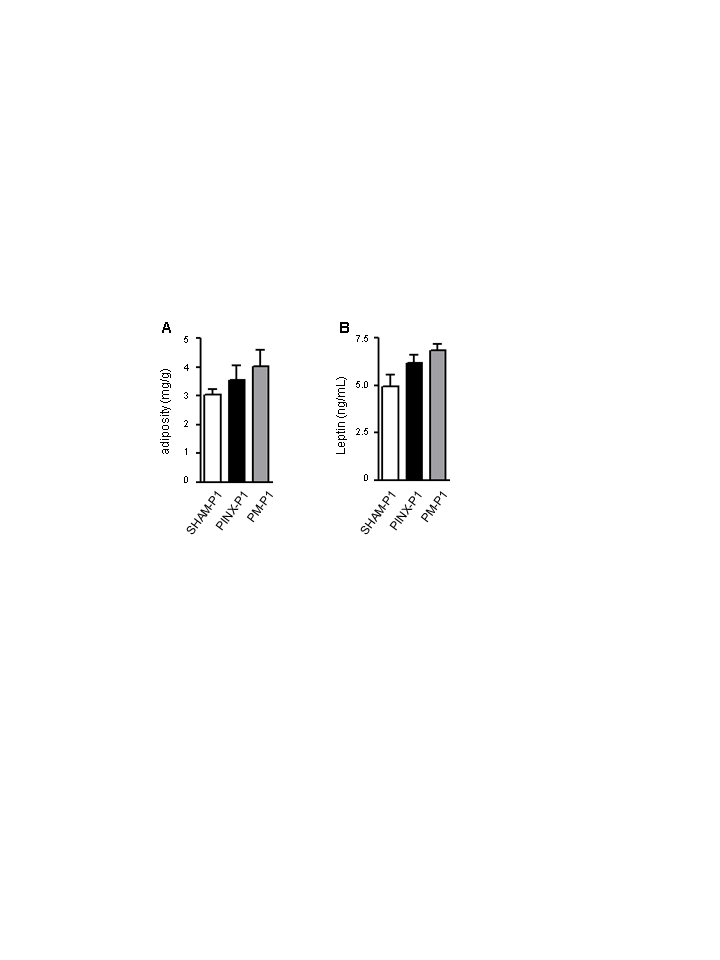
**

Supplement: Figure S1 — Adiposity and serum leptin levels in SHAM-P1, PINX-P1 and PM-P1 rats. Female offspring were anesthetized and weighted at ZT10. Periovarian fat pads were removed and weighted. Adiposity was expressed as the periovarian fat pad mass relative to the rat mass (A). Male rats were anesthetized and decapitated for removal of trunk blood at ZT10. Serum was extracted and processed for leptin determination by ELISA (B). Open bars are SHAM-P1, black bars are PINX-P1 and grey bars are PM-P1. Data are presented as mean ± SE (N=4 to 7 for adiposity; N=5 for leptin measurement). (DOC) [file pone.0038795.s001.doc]

**Figure S2**

**
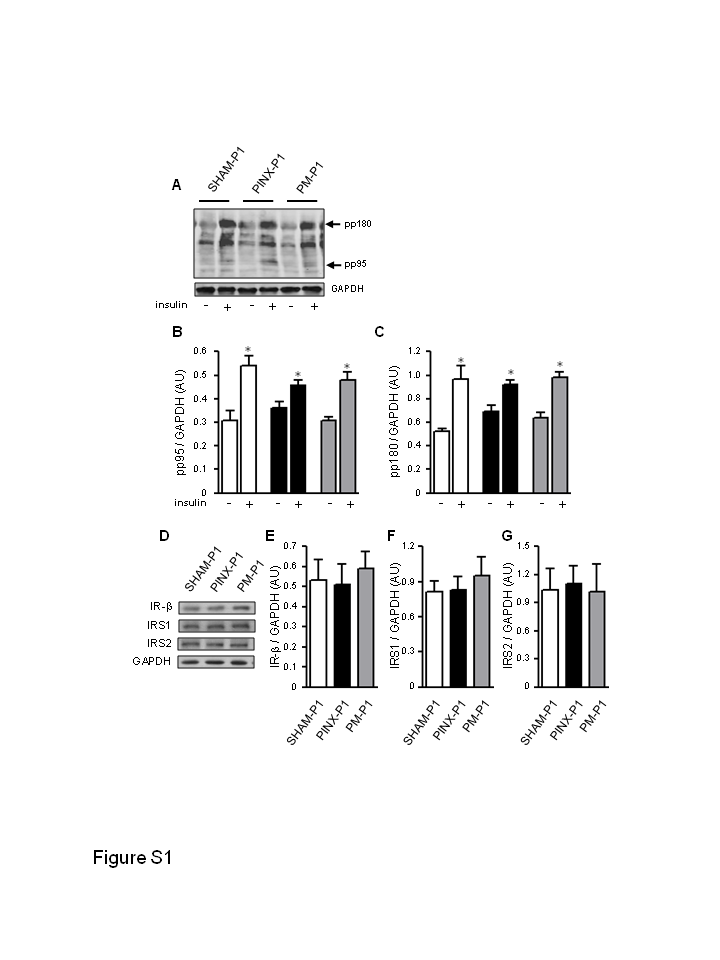
**

Supplement: Figure S2 — Insulin signaling in liver of male SHAM-P1, PINX-P1 and PM-P1. Male offspring were anesthetized at ZT10 and a fragment of the liver was removed to detected basal phosphorylation. An additional fragment of the liver was removed 30 seconds after an intravenous insulin injection. Samples were processed protein extraction and western blot detection of pTyr (A), IR-β, IRS1, IRS2 and GAPDH (D). Values of tyrosine phosphorylated pp95 (B) and pp185 (C) and IR-β (E), IRS1 (F) and IRS2 (G) were normalized to GAPDH Open bars are SHAM-P1, black bars are PINX-P1 and grey bars are PM-P1. Data are presented as mean ± SE. *P<0.05 vs. non-stimulated within the same group (N=6). (DOC) [file pone.0038795.s002.doc]

**Figure S3**

**
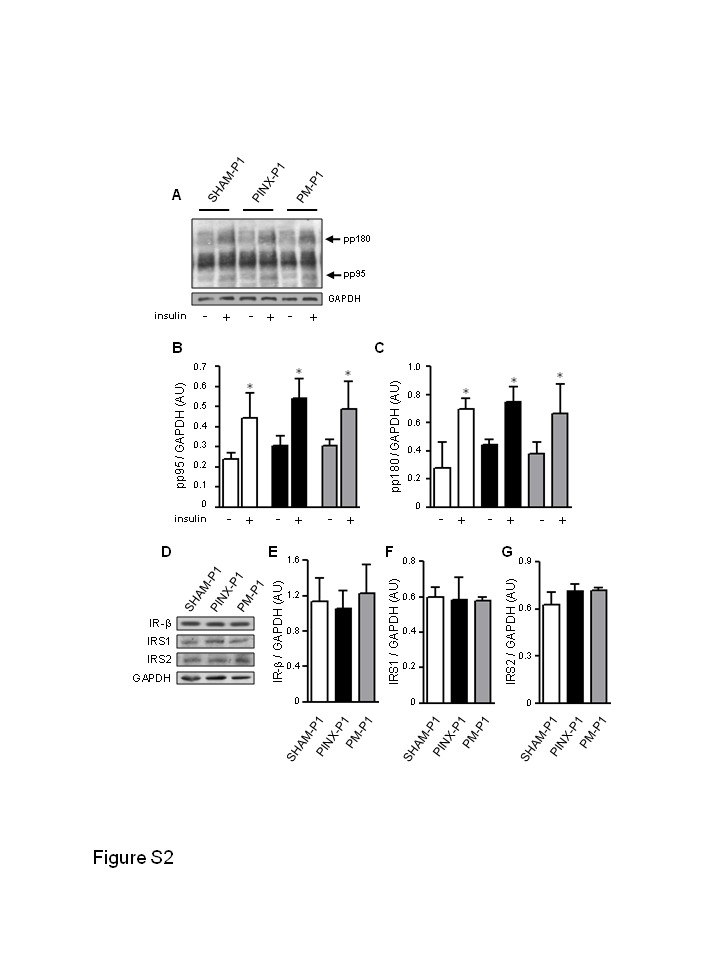
**

Supplement: Figure S3 — Insulin signaling in liver of female SHAM-P1, PINX-P1 and PM-P1. Female offspring were anesthetized at ZT10 and a fragment of the liver was removed to detected basal phosphorylation. An additional fragment of the liver was removed 30 seconds after an intravenous insulin injection. Samples were processed protein extraction and western blot detection of pTyr (A), IR-β, IRS1, IRS2 and GAPDH (D). Values of tyrosine phosphorylated pp95 (B) and pp185 (C) and IR-β (E), IRS1 (F) and IRS2 (G) were normalized to GAPDH Open bars are SHAM-P1, black bars are PINX-P1 and grey bars are PM-P1. Data are presented as mean ± SE. *P<0.05 vs. non-stimulated within the same group (N=6). (DOC) [file pone.0038795.s003.doc]

**Figure S4**

**
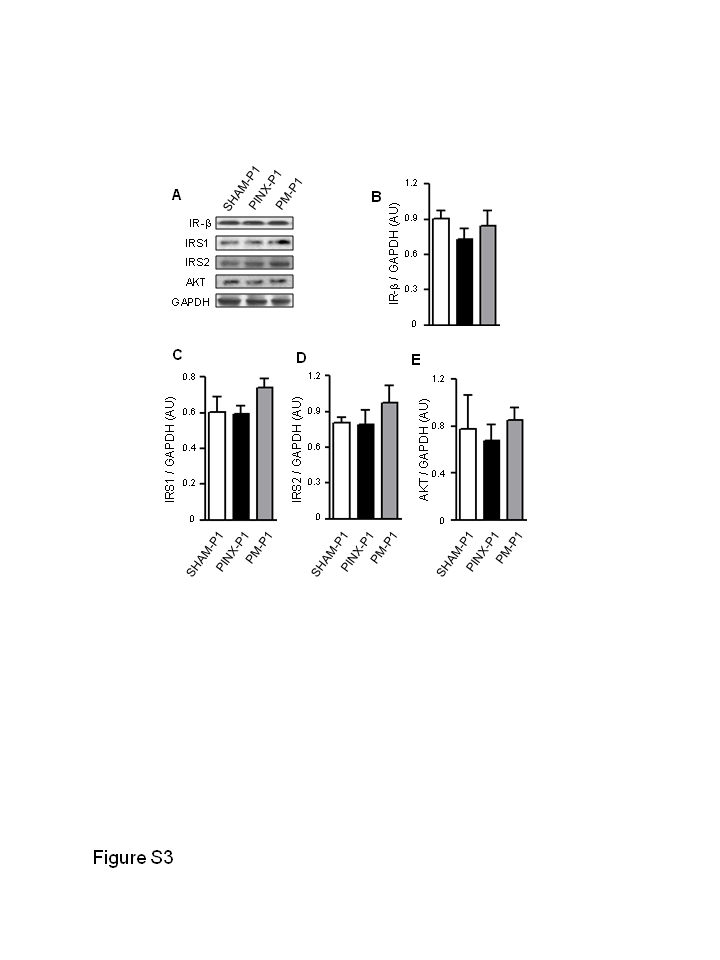
**

Supplement: Figure S4 — Insulin signaling in skeletal muscle of male SHAM-P1, PINX-P1 and PM-P1. Male offspring were anesthetized at ZT10 and a soleus skeletal muscle was removed and processed protein extraction and western blot detection of IR-β, IRS1, IRS2, AKT and GAPDH (A). Values of IR-β (B), IRS1 (C), IRS2 (D) and AKT (E) were normalized to GAPDH. Open bars are SHAM-P1, black bars are PINX-P1 and grey bars are PM-P1. Data are presented as mean ± SE (N=6). (DOC) [file pone.0038795.s004.doc]
